# Pseudopterosin A: Protection of Synaptic Function and Potential as a Neuromodulatory Agent

**DOI:** 10.3390/md14030055

**Published:** 2016-03-10

**Authors:** Stacee Lee Caplan, Bo Zheng, Ken Dawson-Scully, Catherine A. White, Lyndon M. West

**Affiliations:** 1 scaplan4@fau.eduken.dawson-scully@fau.edu; 2 bozhengjob@gmail.comcwhite@rx.uga.edu; 3

**Keywords:** *Pseudopterogorgia elisabethae*, octocoral, pseudopterosins, oxidative stress, *Drosophila melanogaster*, blood-brain barrier, neuromodulatory agent

## Abstract

Natural products have provided an invaluable source of inspiration in the drug discovery pipeline. The oceans are a vast source of biological and chemical diversity. Recently, this untapped resource has been gaining attention in the search for novel structures and development of new classes of therapeutic agents. Pseudopterosins are group of marine diterpene glycosides that possess an array of potent biological activities in several therapeutic areas. Few studies have examined pseudopterosin effects during cellular stress and, to our knowledge, no studies have explored their ability to protect synaptic function. The present study probes pseudopterosin A (PsA) for its neuromodulatory properties during oxidative stress using the fruit fly, *Drosophila melanogaster*. We demonstrate that oxidative stress rapidly reduces neuronal activity, resulting in the loss of neurotransmission at a well-characterized invertebrate synapse. PsA mitigates this effect and promotes functional tolerance during oxidative stress by prolonging synaptic transmission in a mechanism that differs from scavenging activity. Furthermore, the distribution of PsA within mammalian biological tissues following single intravenous injection was investigated using a validated bioanalytical method. Comparable exposure of PsA in the mouse brain and plasma indicated good distribution of PsA in the brain, suggesting its potential as a novel neuromodulatory agent.

## 1. Introduction

Oxygen is essential for the survival of most organisms. A natural byproduct of cellular respiration is the generation of toxic partially reduced forms of oxygen, known as reactive oxygen species (ROS) [1]. ROS is a general term used to describe small molecules containing oxygen that are free radicals or oxidizing agents that are easily converted into radicals. ROS display a variety of biological properties and are known for their dual nature as being both beneficial and deleterious species [2]. ROS serve important roles in cellular signaling pathways, modulate synaptic transmission and plasticity [3], and contribute to cardioprotection resulting from ischemic preconditioning [4]. However, when the concentration of ROS is too high within cells, these molecules can damage cells by disrupting intracellular components such as DNA [5], lipids [6], and proteins [7], affecting cellular membrane permeability, and contributing to the physiology of age-associated disorders [8]. Within biological systems there is a homeostatic balance between the production of ROS and cellular antioxidant defense mechanisms and DNA repair mechanisms that fix the damage. When this dynamic becomes unbalanced and there is an overabundance of ROS, it leads to a cellular condition termed oxidative stress. Oxidative stress is inherent in the pathophysiology of an array of devastating human ailments including ischemic vascular diseases, heart failure, myocardial infarction, stroke, cancer, and numerous neuropsychiatric and neurodegenerative disorders [9,10,11,12]. Few treatment options exist for many of these conditions and more effective strategies could be developed by identifying natural products as novel drug targets and/or therapeutic agents.

**Figure 1:**
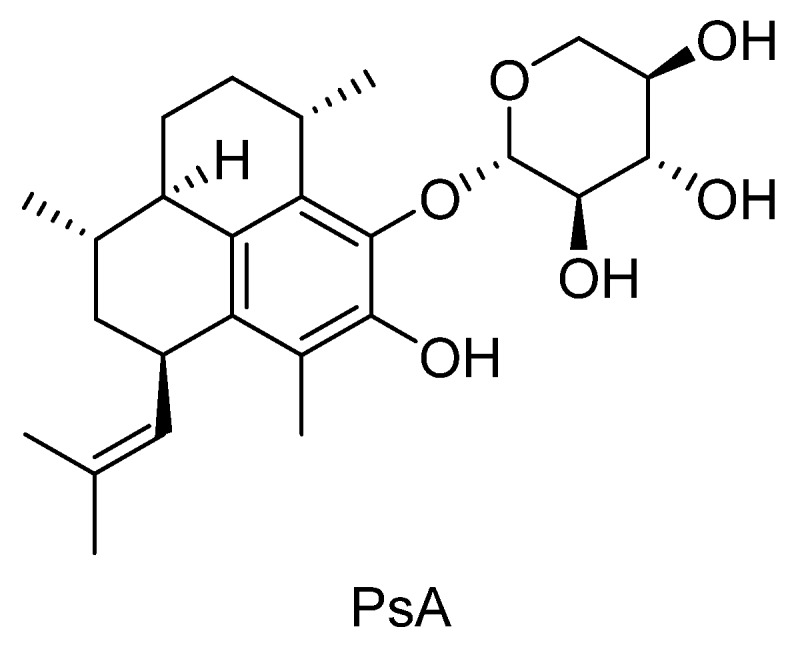
Chemical structure of pseudopterosin A (PsA).

Pseudopterosins are a group of marine diterpene glycosides isolated from the gorgonian soft coral, *Pseudopterogorgia elisabethae* [13,14,15,16]. Thirty-one structurally unique pseudopterosin derivatives have been identified based upon three different isomeric aglycone skeletons [17]. Structural differences among pseudopterosins, such as the position of glycosylation on the terpene skeleton and the type of sugar moiety, affect their biological and cytotoxic activities [18,19]. These compounds possess an array of potent biological activities including anti-inflammatory and analgesic [13,14,20,21,22,23], wound-healing [24,25], anti-bacterial [18,26], anti-cancer, anti-viral, anti-malarial, and anti-tuberculosis [19] in both *in vitro* and *in vivo* assays with a novel mechanism of action [13,14,15,16,25]. They have shown efficacy in Phase II clinical trials as an anti-inflammatory and wound healing agent [26] and are the first commercially licensed natural product for use as an additive in Estée Lauder skin care and cosmetics products [27], which are commercially harvested from a natural, renewable source. Pseudopterosin A (PsA), which contains a non-acetylated xylose sugar subunit (Figure 1), is one of the most extensively studied pseudopterosins and exhibits cell membrane stabilization properties with a novel mechanism of action [28]. PsA has also been shown to alter intracellular calcium and inhibit phagocytosis in free living ciliates and to reduce oxidative bursts during cellular stress in unicellular protists with a unique mode of action [29,30]. Few studies have examined PsA’s effects during cellular stress and, to our knowledge, no studies have explored its potential as a novel neuromodulatory agent.

To determine whether PsA has a neuromodulatory effect during oxidative stress, the present study analyzed PsA’s ability to alter synaptic transmission at the larval neuromuscular junction (NMJ) in the fruit fly, *Drosophila melanogaster*. Invertebrate model systems have helped pave the way to understanding key aspects of the mammalian central nervous system including synapse structure, function, and regulation [31,32]. The *Drosophila* larval NMJ is a well-characterized model for studying the cellular mechanisms of synaptic development and neurotransmission [33,34,35,36]. Oxidative stress was mimicked pharmacologically using two paradigms that generate physiologically relevant oxidant species: mitochondrial superoxide production induced by sodium azide (NaN_3_) and hydroxyl radical formation via hydrogen peroxide (H_2_O_2_). NaN_3_ induces ischemic stress by inhibiting cytochrome c oxidase [37], ATP production [38], superoxide dismutase [39], DNA synthesis, and cell division [40]. H_2_O_2_ propagates hydroxyl radicals by reacting with transition metals [41]. Furthermore, to explore the potential for PsA as a novel neuromodulatory agent, its distribution within biological tissues and ability to cross the blood-brain barrier (BBB) was quantified in a mouse model.

## 2. Results and Discussion

**Figure 2:**
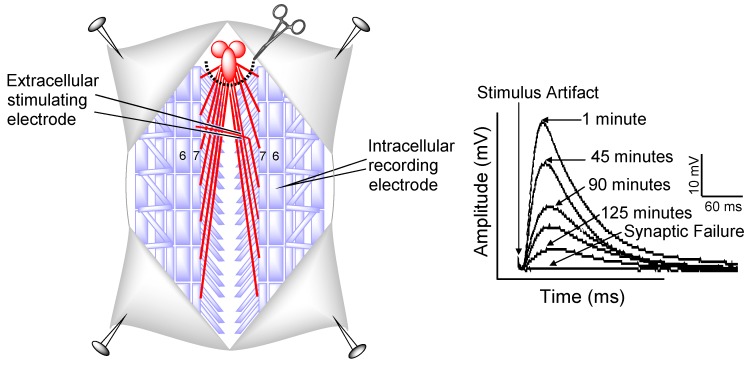
The *Drosophila* larval NMJ preparation and electrophysiological recording of the postsynaptic response. (**Left**) A diagram of the body wall muscles and innervating nerves in 3rd instar *Drosophila* larvae. The larval NMJ dissection technique removes the central nervous system. A presynaptic nerve is suctioned into an extracellular stimulating electrode and the postsynaptic excitatory junction potential (EJP) is recorded from muscle 6/7 with an intracellular electrode. (**Right**) An example of the postsynaptic EJP and its decline over time until synaptic failure (amplitude < 1 mV).

Though their mechanism of action remains unknown, pseudopterosins have been analyzed as novel anti-inflammatory, analgesic, wound-healing, antimicrobial, and anticancer agents [13,14,15,16,18,19,20,21,22,23,24,25,26]. Since few studies have investigated pseudopterosin’s effects during cellular stress, the present study examined the ability of PsA to modulate synaptic function during oxidative stress and quantified its distribution within mammalian biological tissues. The *Drosophila* larval NMJ physiological preparation was used to record synaptic transmission and measure changes in neuronal activity (Figure 2). Oxidative stress was induced pharmacologically using two paradigms: mitochondrial inhibition using NaN_3_ (75 µM) and oxidative overload with H_2_O_2_ (1 mM).

**Figure 3:**
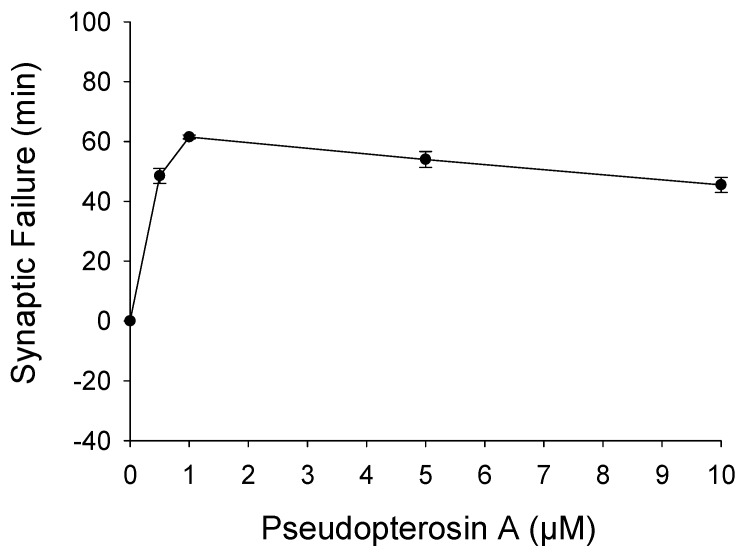
Dose–response curve of pseudopterosin A (0.5–10 µM) during oxidative stress induced by H_2_O_2_ (1 mM) in wild-type Canton-S *Drosophila* larvae.

**Figure 4:**
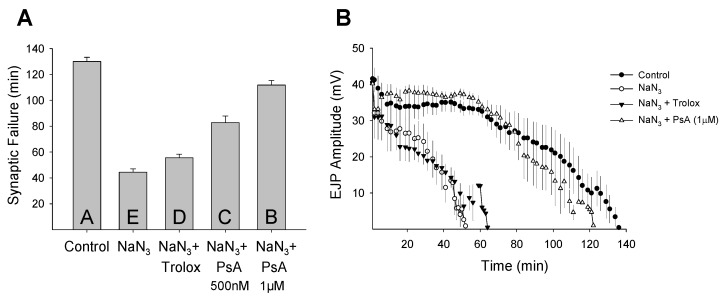
Pseudopterosin A protects synaptic function during NaN_3_ exposure. (**A**) Synaptic transmission at the *Drosophila* larval NMJ failed rapidly during NaN_3_ exposure (75 µM; *N* = 5) compared to control preparations (*N* = 4). Pseudopterosin A (PsA; 500 nM (*N* = 5) or 1 µM (*N* = 4)) and Trolox (5 µM; *N* = 4) showed extension of neurotransmission during the insult (one-way ANOVA, *F*_(4,17)_ = 92.985, *p* < 0.001). Letters in histogram bars represent statistical rankings using a pair-wise multiple comparison procedure, where different letters are statistically significant (Holm-Šidák, *p* < 0.05). All vertical bar charts are shown as means ± SE. (**B**) Amplitude of evoked EJPs reduced over time at different rates in control preparations (saline only) and those exposed to NaN_3_ (75 µM). The addition of either Trolox (5 µM) or PsA (1 µM) to the saline/NaN_3_ bath solution extended the time that synaptic transmission continued.

**Figure 5:**
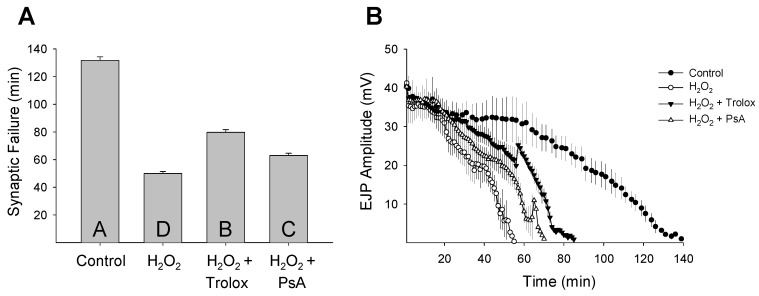
Pseudopterosin A protects synaptic function during H_2_O_2_ exposure. (**A**) Synaptic transmission at the *Drosophila* larval NMJ failed rapidly during H_2_O_2_ exposure (1 mM; *N* = 4) compared to control preparations (*N* = 4). Pseudopterosin A (PsA; 1 µM (*N* = 5)) and Trolox (5 µM; N = 4) showed significant extension of neurotransmission during the insult (one-way ANOVA, *F*_(3,13)_ = 349.27, *p* < 0.001). Letters in histogram bars represent statistical rankings using a pair-wise multiple comparison procedure, where different letters are statistically significant (Holm-Šidák, *p* < 0.05). All vertical bar charts are shown as means ± SE. (**B**) Amplitude of evoked EJPs reduced over time at different rates in control preparations (saline only) and those exposed to H_2_O_2_ (1 mM). The addition of either Trolox (5 µM) or PsA (1 µM) to the saline/H_2_O_2_ bath solution extended the time that synaptic transmission continued.

Organisms respond differently to stress and the cellular basis for stress resistance is still poorly understood. The mammalian brain is especially sensitive to ROS-induced oxidative stress and irreversible damage and cell death can occur within minutes of uncontrolled oxygen fluctuations [42]. Unlike mammals, insects can survive in a critically low oxygen environment for hours without pathology [43,44]. Invertebrate stress resistance mechanisms are believed to have evolved very early and be highly conserved [45]. Previously, we demonstrated that oxidative stress rapidly reduces neuronal function at the *Drosophila* larval NMJ and that this disruption can be protected by pharmacological manipulations [46]. To determine if PsA has a neuromodulatory effect during oxidative stress, we analyzed its ability to alter synaptic function at the *Drosophila* larval NMJ. A dose-response assay from 0.5 to 10 µM was performed to determine the effective concentration of PsA to prolong synaptic transmission during oxidative stress exposure without detrimentally affecting the characteristic features of the evoked postsynaptic response (Figure 3). The *Drosophila* larval NMJ is a well-characterized model for studying neurotransmission with well-documented features [33,34,35,36]; therefore, potential toxicity issues can be detected by a number of factors including changes in the EJP amplitude height, latency time, depolarization and repolarization slopes, as well as the overall shape. Synaptic transmission rapidly declined during both oxidative stress models (NaN_3_ or H_2_O_2_) and simultaneous exposure to Trolox or PsA extended neurotransmission during both insults (Figure 4 and Figure 5). Control preparations exhibited synaptic activity for an average of 130 ± 3 min and NaN_3_ (75 µM) exposure reduced this time frame to an average of 44 ± 3 min (Figure 4A). Larvae treated with Trolox (5 µM) or PsA (1 µM) demonstrated elevated synaptic tolerance during NaN_3_-induced oxidative stress by increasing the time until synaptic failure occurred at 55 ± 3 or 112 ± 4 min, respectively. Similar to what was observed during NaN_3_ treatment, H_2_O_2_ (1 mM) reduced the time until synaptic transmission failure from 132 ± 3 min in controls to 50 ± 2 min (Figure 5A). Simultaneous treatment with Trolox (5 µM) or PsA (1 µM) extended this time frame to 80 ± 2 or 63 ± 2 min, respectively.

In comparison to the well-known antioxidant Trolox [47], PsA demonstrated differential effects depending on the method of oxidative stress induction. During NaN_3_-induced ischemic stress, PsA prolonged synaptic transmission at higher levels than Trolox. PsA (1 µM) increased synaptic functional tolerance by 152%, while Trolox (5 µM) increased synaptic activity by 25% during NaN_3_ exposure. Interestingly, PsA promoted synaptic function less effectively than Trolox during H_2_O_2_-induced oxidative overload (Figure 5). During H_2_O_2_ (1 mM) exposure, Trolox (5 µM) increased synaptic function by 60% and PsA (1 µM) elevated synaptic tolerance by 26%. Mechanistically, Trolox has been shown to protect cells from oxidative damage through scavenging activity [48,49], and these data confirm the well-established antioxidant properties of Trolox. Additionally, these data suggest that PsA acts in a mechanism that is distinct from antioxidant activity and agrees with previous findings that direct scavenging is not the primary mechanism of action of pseudopterosins [29]. It has been suggested that the pseudopterosin site of action may occur at cellular membrane receptors, such as G protein coupled receptors [29,30]. Elucidating the pseudopterosin mechanism of action could be possible using the expansive *Drosophila* toolkit to target specific genes, pathways, and cell surface receptors in many cell types using commercially available fly stocks (*i.e.*, genetic mutants and RNA interference RNAi lines).

Similar to what was observed in the average synaptic failure rates, when the EJP amplitude decline was plotted as a function of time it further demonstrated the difference between Trolox and PsA-induced extension of neurotransmission during oxidative stress (Figure 4B and Figure 5B). Control preparations not exposed to oxidative stress exhibited synaptic activity for maximal times of 136 or 139 min before failure occurred. Oxidative stress induced by either NaN_3_ or H_2_O_2_ reduced this timeframe to 52 or 55 min, respectively. Co-application of Trolox during both stresses increased the time until synaptic breakdown to 64 min during NaN_3_ treatment or 85 min during H_2_O_2_ exposure. Similarly, PsA slowed the rate of amplitude decay and extended the maximal failure time to 122 or 70 min during NaN_3_- or H_2_O_2_-induced oxidative injury, respectively. PsA-induced extension of neuronal function during NaN_3_ exposure persisted at lower concentrations (500 nM), where synaptic tolerance was still elevated by 87% with failure occurring at an average of 83 ± 5 min. These results demonstrate that PsA is able to modulate neuronal activity during oxidative stress and mediate synaptic transmission tolerance by extending the time until neurotransmission breakdown. Since the structure-activity relationships of pseudopterosins are not fully understood, future work will investigate whether other pseudopterosin analogues can modulate synaptic function during oxidative stress and if this ability acts differentially depending on the type of induced stress.

To examine PsA’s biodistribution and ability to access *in vivo* neuronal targets, a bioanalytical method was developed to determine PsA concentration in the mouse plasma, liver, brain, and kidney. Due to the weak UV absorption of PsA, an HPLC method using NBD-Cl as a pre-column derivatization reagent was developed to increase the UV absorption of PsA. 17β-estradiol was used as the internal standard (IS) for method validation (see Figure S1 in the Supplementary Information). To optimize this derivatization procedure, reaction conditions (*i.e.*, presence or absence of catalyst, temperature, and reaction time) were varied and the reaction mixtures were analyzed by HPLC. The optimum reaction parameters were determined to be 30 min, with 18-crown-6 and potassium carbonate. The LLOQ of PsA using this method was 0.05 µg/mL or µg/g in biological tissues (see Figures S2–S5 in the Supplementary Information). For validation, the linearity, precision, and accuracy of this method were analyzed. A linear relationship (*R*^2^ > 0.998) over the range of 0.05–60 µg/mL PsA in the mouse plasma, brain, liver, and kidney was found, which indicated high precision (see Table S1 in the Supplementary Information). The absolute recoveries of PsA ranged from 93.8% to 98.8% (see Table S2 in the Supplementary Information). The precision and accuracy for intra- and inter-day PsA measurements were less than 10%, demonstrating the reliability and reproducibility of this method (see Table S3 in the Supplementary Information).

**Figure 6:**
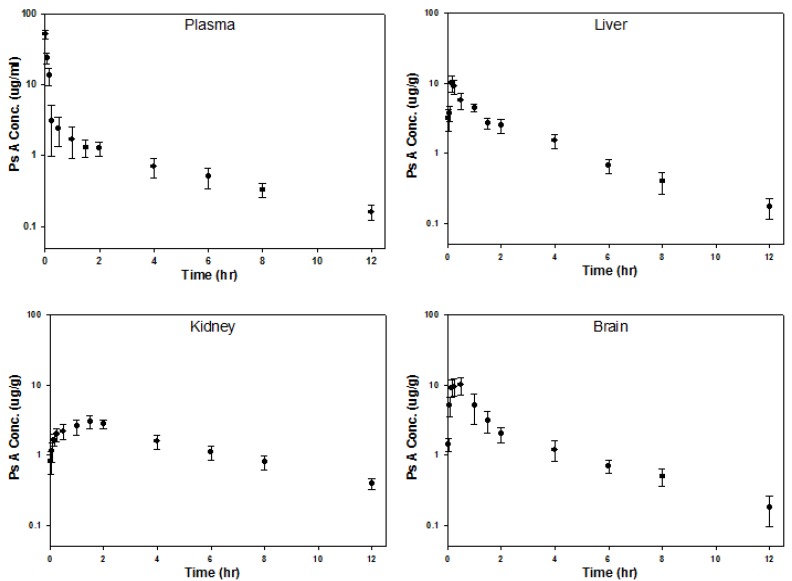
Concentration *vs.* time profiles of PsA in the plasma, liver, brain, and kidney after 50 mg/mL iv bolus doses of PsA via the tail vein in male NIH Swiss mice (25–30 g). The mice were fasted for 12 h before receiving PsA and fed 4 h after drug administration. The mice (*N* = 3 per time point) were sacrificed under anesthesia and blood and tissue samples were collected at each time point (2, 5, 10, 15, 30, 60, 90, 120, 180, 240, 360, 480, 720 min) into heparinized microcentrifuge tubes.

PsA distribution in mammalian tissues following a 50 mg/kg intravenous injection was quantified using the abovementioned bioanalytical method. This dose was well tolerated in mice. Water and food intake and general appearance of the mice were normal during the study. No signs of toxicity (*i.e.*, lethargy, bleeding, muscle spasm, loss of motility, reduced body temperature, agitation, death, *etc.*) were observed in the mice within 12 h post iv dosing. As shown in Figure 6, mean plasma concentration of PsA declined in a bi-exponential fashion post intravenous injection. PsA mean concentration in the liver and brain rapidly reached peak levels of 10.06 µg/g at 10.2 min and 10.02 µg/g at 30 min, respectively, and declined in a bi-exponential fashion, indicating rapid tissue uptake in liver and brain. Interestingly, PsA concentrations in liver and brain were in excess of that in plasma 10–15 min post injection, suggesting high partitioning of PsA in liver and brain. Compared to PsA concentration in the liver and brain, the mean concentration in the kidney peaked much later (90 min) and was considerably lower at 3.00 µg/g. However, PsA mean concentration in the kidney was higher than that observed in plasma and other tissues 90 min post intravenous injection, indicating that the rates of forward and reverse distribution of PsA between plasma and kidney are slower than those in the liver and brain. Furthermore, tissue/plasma concentration ratios of PsA in those three tissues were > 1 during the elimination phase, suggesting accumulation of PsA in the brain, liver, and kidney. This could be attributed, in part, to the high lipophilicity of the compound [50].

Pharmacokinetic (PK) parameters of PsA in mouse plasma and tissues were generated by two-compartmental analysis using the mean concentration at each time point (Table 1 and Table 2; see Figure S6 in the Supporting Information). In plasma, PsA distribution and elimination half-lives were 0.05 and 3.21 h, respectively, indicating that it is rapidly distributed into mouse tissues following intravenous injection. The distribution volume at steady state (*V_ss_*) of PsA was 10.09 L/kg which is larger than the total body water volume (0.43 L/kg) [51], indicating extensive distribution of PsA in mouse tissues. Clearance (CL) of PsA was 3.48 L/h/kg and comparable to the values of blood flow in liver (4.46 L/h/kg) and kidney (2.52 L/h/kg) [52], suggesting that PsA is rapidly cleared in the mouse. In mouse tissues, PsA terminal half-life ranged from 2.91 h (brain) to 3.98 h (kidney), which is comparable to PsA plasma half-life of 3.21 h and suggests a good correlation between plasma and tissue PK. The area under the curve (AUC) for all tissues was larger than the plasma AUC (14.35 h∙mg/L), yielding relative exposure (RE) in the range of 1.27–1.36. Among these tissues, the brain had the largest AUC (19.49 h∙mg/L), indicating that PsA extensively distributes into the brain.

The key PK characteristics of PsA demonstrated by the present study include: (i) PsA is rapidly eliminated in the mouse with an elimination half-life of 3–4 h in the plasma and tissues (brain, liver, kidney) and (ii) distribution of PsA in the brain and other mouse tissues (liver and kidney) is extensive with tissue/plasma concentration ratios of PsA > 1 during the elimination phase. Since the blood-brain barrier (BBB) is the most important barrier impeding drug transport into the brain, the ability to penetrate the BBB or distribute within the brain is a key feature to assess during the development process of CNS drugs. These PK findings suggest the potential of PsA as a novel neuromodulatory agent.

## 3. Experimental Section

### 3.1. Animal Material

The gorgonian *Pseudopterogorgia elisabethae* was collected by hand using SCUBA at a depth of 55 feet from Burrows Cay, Bahamas. The specimen was immediately frozen and kept at −20 °C until extraction. A voucher specimen has been deposited at the Department of Chemistry and Biochemistry, Florida Atlantic University, Boca Raton (BA06-037).

### 3.2. Extraction and Isolation

The soft coral *P. elisabethae* (6 g dry wt.) was minced and extracted with MeOH (3 × 400 mL). The third, second, and then the first extracts were passed through a column of HP-20 resin (2.5 × 25 cm) equilibrated with MeOH. The combined eluents was diluted with H_2_O (3.6 L) and passed again through the column. The column was eluted with 400 mL fractions of (1) H_2_O; (2) 40% Me_2_CO/H_2_O; (3) 75% Me_2_CO/H_2_O and (4) Me_2_CO. Fraction 3 was back-loaded onto an HP-20 column to remove the H_2_O by passing the fraction through a column of HP-20 resin (2.5 × 8.0 cm) equilibrated with H_2_O. The eluent was diluted with H_2_O (500 mL) and passed again through the column. The column was eluted with Me_2_CO (250 mL), and then 50% MeOH/Me_2_CO (250 mL), and the combined fractions concentrated to dryness. Fraction 3 was subjected to preparative C18 reversed-phase HPLC (Gemini 5 μm; 21.2 × 250 mm; 8 mL/min; 75%–100% CH_3_CN/H_2_O over 60 min) to give pseudopterosin A (**1**). The structure was determined by mass spectral data and direct comparison of the ^1^H and ^13^C NMR data to the previously reported NMR data.

### 3.3. Fly Stocks

This study used wild-type Canton-S *Drosophila melanogaster* third instar wandering larvae (~110 h old). All larvae were raised within an equal population density in 170 mL plastic culture bottles (~100 flies per bottle) containing 50 mL of Nutri-Fly Bloomington Formulation (Genessee Scientific, San Diego, CA, USA) fly food in an incubator at 25 °C under a 12 h:12 h light–dark light cycle.

### 3.4. Electrophysiology

Synaptic transmission was measured in Canton-S animals as previously described [46]. Briefly, individual Canton-S third instar larvae were placed in a glass dissecting dish containing 2 mL of Schneider’s insect medium (Sigma, St. Louis, MO, USA) with the dorsal side up using standard insect pins. A longitudinal cut was made along the dorsal surface and the internal organs and central nervous system were removed to expose the underlying segmental muscles and nerves. An extracellular glass suction electrode was used to stimulate segmental nerves in muscle segments. The postsynaptic excitatory junction potential (EJP) was recorded from muscle 6 in abdominal segment A3 or A4 with a sharp intracellular glass recording electrode filled with 3 M KAc (~40 MΩ). The Schneider’s insect medium was replaced with 2 mL of hemolymph-like (HL-3) saline (1.5 mM CaCl_2_, 20 mM MgCl_2_, 5 mM KCl, 70 mM NaCl, 10 mM NaHCO_3_, 5 mM BES, 115 mM sucrose, 5 mM trehalose·2H_2_O) made fresh daily [53,54]. EJP recordings were viewed with an oscilloscope and digitally stored using the Scope program (AD Instruments, Colorado Springs, CO, USA) for analysis. Evoked EJPs from repetitive stimulation (0.3 ms pulses delivered suprathreshold with a 1 Hz frequency) of both axons in larval muscle 6 were recorded in a stop-flow condition until synaptic transmission failure (amplitude < 1 mV).

### 3.5. Pharmacology

The same electrophysiology preparation assay described above was performed; however, Canton-S larvae were also exposed to oxidative stress, Trolox, and PsA. Oxidative stress was induced in larval preparations by adding either 1 mM H_2_O_2_ or 75 µM NaN_3_ to the HL-3 saline (all chemicals obtained from Sigma, St. Louis, MO, USA). Larvae subjected to oxidative stress and simultaneous drug treatment included one of the following agents dissolved in DMSO: 5 µM Trolox or 1 µM PsA. A dose-response assay from 0.5 to 10 µM was performed to determine the effective concentration of PsA to prolong synaptic transmission at the *Drosophila* larval NMJ during oxidative stress exposure (Figure 3). A similar concentration of Trolox was used to enable accurate comparisons of synaptic failure times between drug treatments.

### 3.6. Drosophila NMJ Electrophysiology Statistics

All synaptic function data were analyzed using SigmaPlot (Systat Software, San Jose, CA, USA) statistical analysis software. A one-way analysis of variance (ANOVA) test followed by a *post hoc* multiple comparisons procedure (Holm–Šidák) was used to compare significant differences across all treatment groups. Statistical significance (*p* < 0.05) was assigned using letter designations, where different letters show significant differences and the same letter assignments are not significant. The letter assignments begin with “A” representing the highest mean, “B” indicating the next highest, and so forth. All vertical bar charts are shown with the means ± SE.

### 3.7. Preparation of Stocks, Calibration Standards, and Quality Control Samples

Stock solutions of 1.0 mg/mL PsA, 17β-estradiol and 5.0 mg/mL 4-chloro-7-nitrobenzofurazan (NBD-Cl) were individually prepared in acetonitrile (all chemicals obtained from Sigma). Standard solutions of PsA were prepared by mixing and diluting the appropriate amounts from the individual stock solutions. The final concentrations of the standard solutions were 600, 200, 100, 50, 10, 5, and 0.5 µg/mL. A 2.5 mg/mL standard solution of 4-chloro-7-nitrobenzofurazan was prepared with acetonitrile from 5 mg/mL stock. A 100 µg/mL standard solution of 17β-estradiol was prepared with acetonitrile from 1 mg/mL stock. The quality control samples (QCs) were prepared in duplicate from separate stock solutions. High-, mid-, and low-level quality control samples contained 400, 10, and 1 µg/mL of PsA were prepared in a manner similar to that used for the preparation of the calibration standards. Stock solutions were kept refrigerated when not in use and replaced on a bi-weekly basis. Fresh standard solutions were prepared for each day of analysis or validation.

### 3.8. Calibration Curves

Blank plasma, liver, brain, kidney tissue were collected from untreated anesthetized mice. The tissues were homogenized with same volumes of deionized water (*w*/*v*) using an Ultra-Turbax T8 tissue grinder (IKA Labortechnik, Staufen, Germany). Plasma calibration points were prepared by spiking 100 µL of the biological matrices with 10 µL of each PsA and 17β-estradiol standard solution. Tissues calibration points were prepared by spiking 200 µL of the biological matrices with 10 µL of each PsA and 17β-estradiol standard solution. The calibration curves of all matrices were in the range of 0.05–60 µg/mL, with an internal standard concentration in each sample of 10 µg/mL. After each matrix was spiked, it was subject to further sample preparation before analysis.

### 3.9. Sample Preparation

After spiking with 10 µL of 17β-estradiol (100 µg/mL), 100 µL plasma or 200 µg of tissue homogenate (prepared as above) was mixed with 300 µL of acetonitrile and vortex-mixed for 15 s. The samples were then extracted with 0.6 mL of ethyl acetate. After centrifugation for 10 min at 2000× *g*, the upper organic layer was removed and evaporated to dryness under reduced pressure in a vacuum centrifuge. The residue obtained was reconstituted in 80 µL acetonitrile with ultrasonic treatment for 1 min and the derivatization was performed as follows.

### 3.10. Liquid Chromatography

The HPLC measurements were carried out using a Shimadzu LC-20AT liquid chromatography, which were coupled to a UV-SPD-M20A diode array detector. Data acquisition was performed using EZStart chromatography software package version 7.4. The chromatographic separation of NBD-Cl -derivative of PsA was achieved using Phenomenex Gemini 5 µm C18 110 Å column with a Phenomenex Security Guard C18 guard column (Torrance, CA, USA). The Mobile phase: A: 20 mM AcONH_4_ (pH = 7.4): ACN (2:1); B: CAN. Running program: 0–5 min, 80%–90% B; 5–7 min, 90% B; 7–12 min, 90%–100% B; 12–15 min, 100%–80% B. The mobile phase flow rate was 1.0 mL/min and the UV detection wavelength was set to 378 nm. Under the chromatographic conditions described, NBD-Cl derivatives of PsA and 17β-estradiol eluted at 7.3 and 5.5 min, respectively.

### 3.11. Linearity, Precision, and Accuracy of the Bioanalytical Method

The linearity of the HPLC method for the determination of PsA was evaluated by a calibration curve in the range of 0.05–60 µg/mL or µg/g. The calibration curve was obtained by plotting the ratio of peak area of each analyte to internal standard *versus* PsA theoretical concentration. Least squares linear regression analysis was used to determine the slope, intercept, and correlation coefficient. The calibration curve required a correlation coefficient (*r*^2^) of 0.99 or better, which was considered appropriate for a validated method. To evaluate precision, at least five QCs at each of the three different concentrations were processed and injected on a single day (intra-day) and on different days (inter-day). The variability of PsA determination was expressed as the coefficient of variation (% CV), which should be ≤15% for all concentrations. Accuracy was expressed as % bias of theoretical *versus* calculated concentrations, and it should be within limits of ±15% for all concentrations of PsA.

### 3.12. Recovery

The absolute recoveries of PsA from plasma and tissues were determined at different standard concentrations by spiking the drug into the corresponding fresh blank plasma or tissues. The percentage of recovery was calculated by comparing the peak area of extracted samples with samples in which the same amount of compounds were diluted with acetonitrile, taken through a derivatization reaction, and injected directly. The recoveries at three QC concentration levels of PsA in plasma and tissues were examined at least five times.

### 3.13. Pharmacokinetic Experiments in Mice

Male NIH Swiss mice (25–30 g) were obtained from *Harlan* Laboratories, Inc. The mice were acclimated (5 mice/cage) for at least 7 days in an AAALAC (Association for Assessment and Accreditation of Laboratory Animal Care) approved animal care facility after arrival. All experimental protocols were approved by the Laboratory Animal Care and Use Committee at the University of Georgia. The mice were fasted 12 h before receiving PsA and fed 4 h after drug administration. All mice received an IV bolus dose of PsA (50 mg/kg) via the tail vein. The mice (*N* = 3 per time point) were sacrificed under anesthesia and blood samples were collected at each time point (2, 5, 10, 15, 30, 60, 90, 120, 180, 240, 360, 480, 720 min) into heparinized microcentrifuge tubes. Plasma was prepared by centrifugation of blood at 1000 *g* for 15 min at 4 °C and stored at −80 °C until analysis. Tissues (brain, liver, and kidney) were also collected and homogenized (with 1:1 water for tissues) and stored at −80 °C until analysis. Plasma, liver, brain, and kidney were processed and analyzed as mentioned above. A calibration curve from each matrix was prepared on the day of analysis to calculate the concentrations of PsA present in the real samples. The concentration–time profiles of PsA in all matrices were plotted. The plasma data was subjected to compartmental analysis using *WinNonlin* 5.2. (Pharsight, Mountain View, CA, USA). A two-compartment intravenous bolus model with first-order elimination was used to fit the plasma data. A 1/*y*^2^-weighting scheme was used throughout the analysis. Pharmacokinetic parameters such as half-life, volume of distribution, clearance, and area under curve were determined. Pharmacokinetic analysis of PsA concentrations in tissues was performed using non-compartmental and compartmental methods using *WinNonlin* 5.2. Pharmacokinetic parameters such as half-life, area under curve (AUC) were determined. Relative exposure (RE) of each tissue was calculated as AUC_tissue_/AUC_plasma_.

## 4. Conclusions

These data demonstrate that PsA alters synaptic activity by promoting function during oxidative stress and readily crosses the BBB, indicating its potential as a novel neuromodulatory agent. Recent evidence suggests that the pseudopterosin site of action may occur at cellular membrane receptors (*i.e.*, ion channels) and their conformational change during oxidation could represent a reversible mechanism for receptor activation/deactivation [55,56]. Additional experiments testing this hypothesis can be readily addressed using the expansive genetic toolkit of the *Drosophila* model system that allow specific genes, pathways, and cell surface receptors to be targeted using commercially available transgenic fly stocks (*i.e.*, genetic mutants and RNA interference (RNAi) lines). Furthermore, the physiochemical properties of PsA allow it to be administered intravenously, which is the preferred route of administration for the treatment acute neurological conditions such as traumatic brain injury and stroke. Investigating different preparations of PsA, such as cyclodextrin formulation, could also be beneficial for use in a clinical setting [57]. Given that many pharmaceuticals have been inspired by natural products and derivatives thereof and that pseudopterosins, in general, have an expansive list of biological activities, these compounds deserve further investigations into their neuroactive properties so that we may shed light on their elusive mechanism of action and harness their therapeutic potential.

## Figures and Tables

**Table 1:** Plasma pharmacokinetic parameters for PsA (estimate ± standard error).

Parameter	Value
Distribution Half-life (h)	0.05 ± 0.005
Elimination Half-life (h)	3.21 ± 0.30
AUC (h·mg/L)	14.35 ± 0.95
CL (L/h·kg)	3.48 ± 0.23
*V_ss_* (L/kg)	10.09 ± 1.35
*C*_max_ (mg/L)	85.49 ± 7.64

**Table 2:** Brain, liver, and kidney pharmacokinetic parameters and relative exposures for PsA.

Parameter	Brain	Liver	Kidney
Half-life (h)	2.91	3.16	3.98
AUC * (h·mg/kg)	19.49	18.20	18.37
*C*_max_ (μg/g)	10.02	10.06	3.00
*T*_max_ (h)	0.5	0.17	1.5
Relative Exposure **	1.36	1.27	1.28

* AUC_last_: Area under the concentration-time curve from time 0 to the time of the last measurable concentration, calculated using the linear trapezoidal rule; ** Relative Exposure = AUC_tissue_/AUC_plasma_.

## Supplementary Materials

The following are available online at http://www.mdpi.com/1660-3397/14/3/55/s1.

## Abbreviations

The following abbreviations are used in this manuscript:

PsA	pseudopterosin A
ROS	reactive oxygen species
NMJ	neuromuscular junction
NaN_3_	sodium azide
H_2_O_2_	hydrogen peroxide
BBB	blood–brain barrier
LLOQ	lower limit of quantification
HPLC	high-performance liquid chromatography
NBD-Cl	4-chloro-7-nitrobenzofurazan
IV	intravenous
AUC	area under the curve
RE	relative exposure
EJP	excitatory junction potential
DMSO	dimethyl sulfoxide
