# Supplementary material for: Pseudopterosin A: Protection of Synaptic Function and Potential as a Neuromodulatory Agent
Source: Mar Drugs. 2016 Mar 10;14(3):55. doi: 10.3390/md14030055 (PMC4820309; doi:10.3390/md14030055)
Supplement: Supplementary file 1 [file marinedrugs-14-00055-s001.pdf]

Supplementary Information: Pseudopterosin A: Protection of Synaptic Function and Potential as a Neuromodulatory Agent

Stacee Lee Caplan, Bo Zheng, Ken Dawson-Scully, Catherine A. White and Lyndon M. West

Table S1. Linear regression equations generated from validation data for each matrix; slope ± S.D., correlation coefficient ± S.D.

Matrix	Slope	R^2
Plasma	8.632 ± 0.52	0.9998 ± 0.07
Brain	8.624 ± 0.78	0.9996 ± 0.07
Liver	8.701 ± 0.92	0.9980 ± 0.10
Kidney	8.543 ± 1.0	0.9984 ± 0.12

Table S2. Absolute recovery (mean ± S.D.) of the method for determining the concentration of PsA in plasma, brain, liver, and kidney (n = 15).

Concentration (μg/mL or μg/g)	Plasma	Brain	Liver	Kidney
0.05	95.51 ± 3.21	94.25 ± 5.78	93.76 ± 5.85	95.02 ± 3.78
0.1	95.77 ± 2.15	94.78 ± 5.49	94.24 ± 3.31	96.13 ± 2.89
1	98.52 ± 3.57	96.33 ± 3.65	95.33 ± 7.12	97.42 ± 3.14
40	98.78 ± 4.76	96.54 ± 4.01	96.13 ± 2.66	98.10 ± 2.05

Table S3. Intra-day (n = 5) and inter-day (n = 15) precision and accuracy of PsA measurement in each matrix.

Biological Matrix	T.C.	Intra-Day			Inter-Day		
		E.C.	Precision (% CV)	Accuracy (% Error)	E.C.	Precision (% CV)	Accuracy (% Error)
Plasma	0.1	0.10	6.64	7.12	0.11	7.91	8.83
	1	1.02	5.35	5.63	1.03	5.23	4.62
	40	41.3	4.98	3.54	41.1	4.05	2.96
Brain	0.1	0.10	5.37	4.73	0.10	4.61	4.81
	1	1.02	4.09	5.25	1.03	4.37	4.89
	40	40.6	5.58	4.39	39.5	3.32	3.21
Liver	0.1	0.10	6.98	5.53	0.11	7.15	4.93
	1	1.02	5.21	5.14	1.04	5.56	5.04
	40	40.6	6.91	4.88	39.7	5.12	4.42
Kidney	0.1	0.11	5.04	6.72	0.10	4.89	3.87
	1	1.01	4.52	5.87	1.02	3.95	5.65
	40	38.52	4.34	3.72	40.5	3.51	3.61

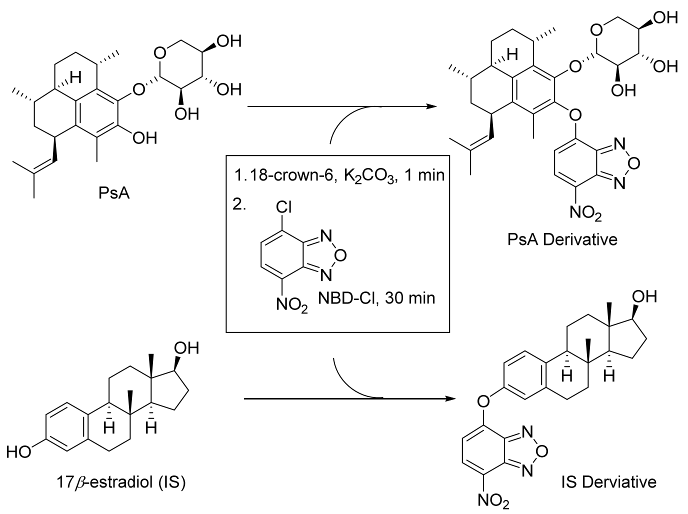

Figure S1. Derivatization scheme for PsA and IS with NBD-Cl.

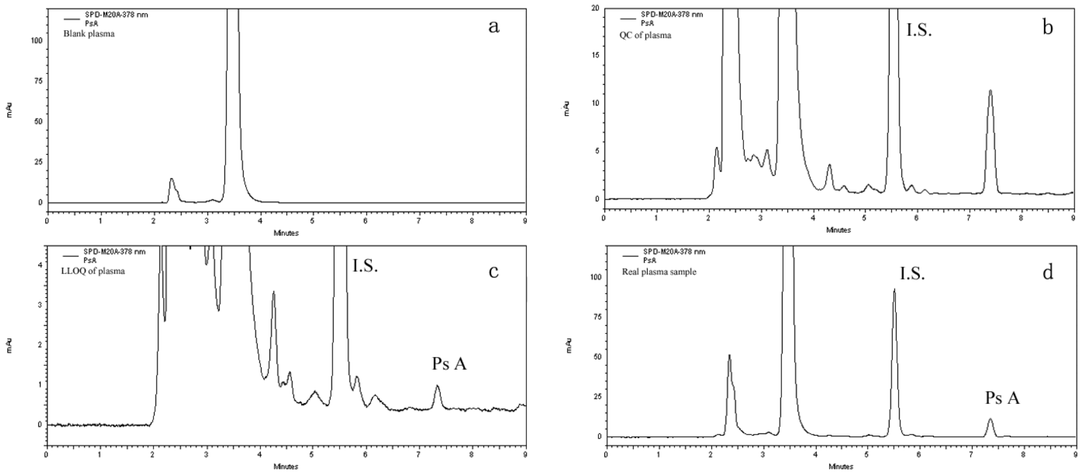

Figure S2. Representative HPLC chromatograms: (**a**) blank plasma sample; (**b**) a 1 μg/mL PsA quality control plasma sample; (**c**) the LLOQ of Ps A (0.05 μg/mL) in plasma; and (**d**) a mouse plasma sample 30 min after a 50 mg/kg dose of PsA.

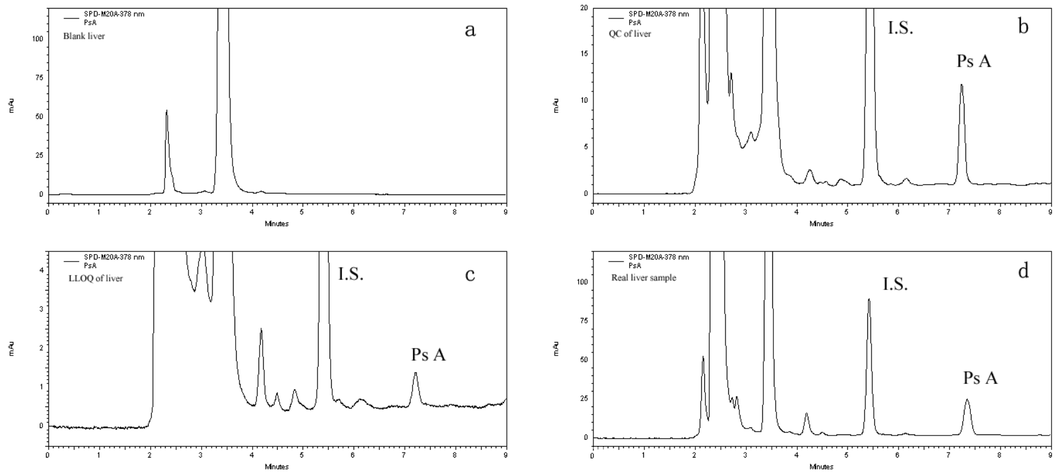

Figure S3. Representative HPLC chromatograms: (**a**) blank liver sample; (**b**) a 1 μg/mL PsA quality control liver sample; (**c**) the LLOQ of PsA (0.05 μg/mL) in liver; and (**d**) a mouse liver sample 30 min after a 50 mg/kg dose of PsA.

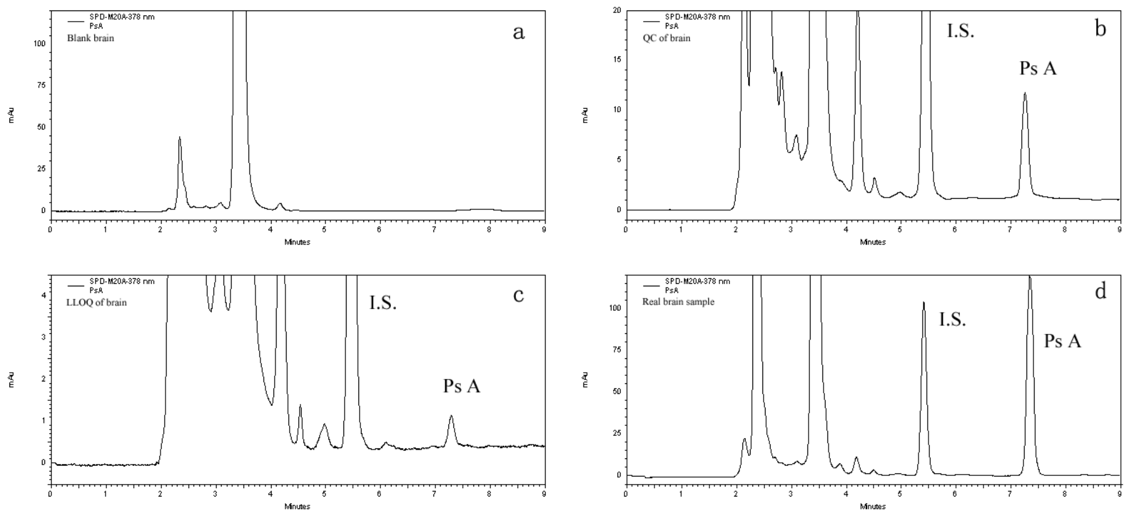

Figure S4. Representative HPLC chromatograms: (**a**) blank brain sample; (**b**) a 1 μg/mL PsA quality control brain sample; (**c**) the LLOQ of PsA (0.05 μg/mL) in brain; and (**d**) a mouse brain sample 30 min after a 50 mg/kg dose of PsA.

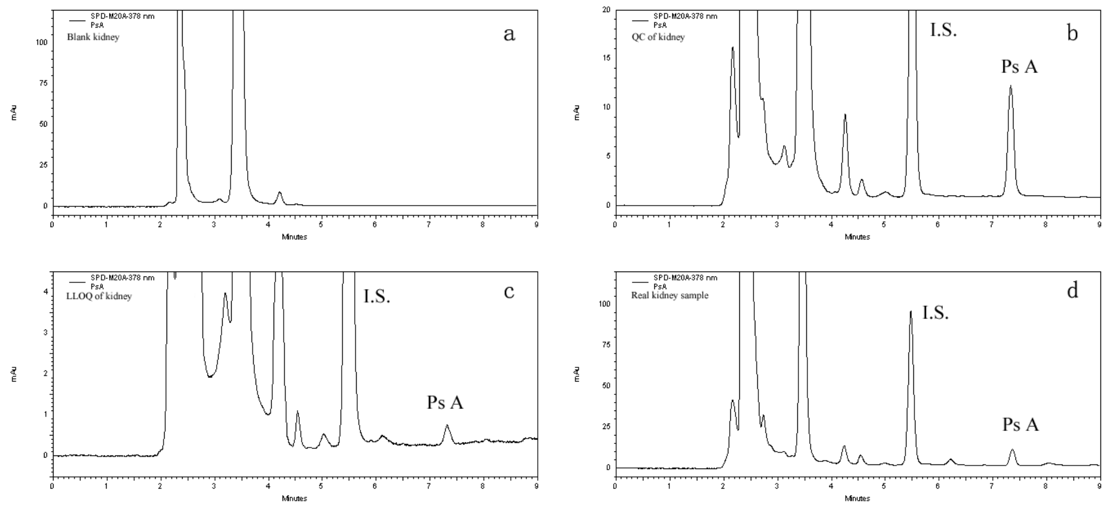

Figure S5. Representative HPLC chromatograms: (**a**) blank kidney sample; (**b**) a 1 μg/mL PsA quality control kidney sample; (**c**) the LLOQ of PsA (0.05 μg/mL) in kidney; and (**d**) a mouse kidney sample 30 min after a 50 mg/kg dose of Ps A.

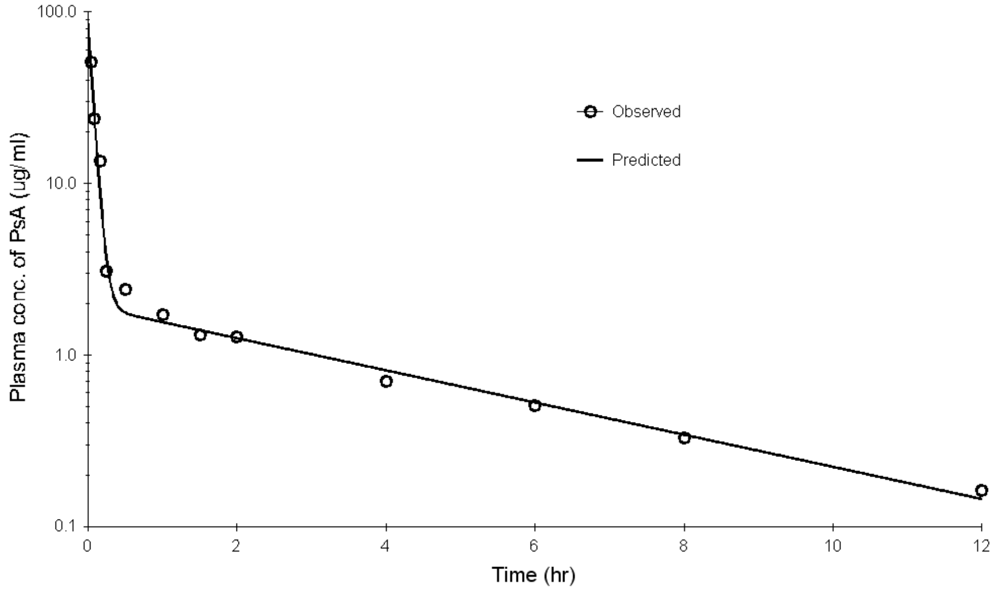

Figure S6. The mean plasma concentration–time profile after iv administration of PsA was fitted to a two-compartment model.
